# Circannual changes in stress and feeding hormones and their effect on food-seeking behaviors

**DOI:** 10.3389/fnins.2013.00140

**Published:** 2013-08-07

**Authors:** Shaina Cahill, Erin Tuplin, Matthew R. Holahan

**Affiliations:** Department of Neuroscience, Carleton UniversityOttawa, ON, Canada

**Keywords:** leptin, glucocorticoids, ghrelin, seasonal variation, stress, feeding

## Abstract

Seasonal fluctuations in food availability show a tight association with seasonal variations in body weight and food intake. Seasonal variations in food intake, energy storage, and expenditure appear to be a widespread phenomenon suggesting they may have evolved in anticipation for changing environmental demands. These cycles appear to be driven by changes in external daylength acting on neuroendocrine pathways. A number of neuroendocrine pathways, two of which are the endocrine mechanisms underlying feeding and stress, appear to show seasonal changes in both their circulating levels and reactivity. As such, variation in the level or reactivity to these hormones may be crucial factors in the control of seasonal variations in food-seeking behaviors. The present review examines the relationship between feeding behavior and seasonal changes in circulating hormones. We hypothesize that seasonal changes in circulating levels of glucocorticoids and the feeding-related hormones ghrelin and leptin contribute to seasonal fluctuations in feeding-related behaviors. This review will focus on the seasonal circulating levels of these hormones as well as sensitivity to these hormones in the modulation of food-seeking behaviors.

## General overview

The purpose of this review is to examine seasonal variation in responses to and circulating levels of glucocorticoids and ghrelin and leptin in relation to their facilitation or attenuation of feeding- and reward-related behaviors during the summer and winter seasons. We became interested in determining whether seasonal fluctuations in either stress or feeding hormones would interfere with these behaviors in the winter or facilitate food- and reward-related behavioral output in the summer. As such, literature will be reviewed that has investigated circannual changes in responses to and circulating levels of glucocorticoids and the feeding hormones, ghrelin and leptin. While others have provided comprehensive reviews on circadian fluctuations in these hormones (Kalra et al., 2003; Engeland and Arnhold, 2005; Lightman, 2008; Dietrich and Horvath, 2009; Papadimitriou and Priftis, 2009; Mistlberger, 2011), this paper reviews literature on the circannual fluctuations in these hormone levels and how they might influence feeding and feeding-related behaviors (such as bar pressing). As well, while there may be interesting relationships between circannual and circadian rhythms in the control of feeding [for a review on this topic in fish, see Volkoff et al. (2010)], a thorough discussion of this topic is beyond the scope of this review paper, though will be touched on briefly. Therefore, this review will examine how otherwise enhanced food-related behaviors might be attenuated by seasonal fluctuations in glucocorticoids and ghrelin and leptin signaling. This review may provide insight into inconsistent findings in experimental literature that might be due, in part, to seasonal variation in these hormones.

### Seasonal variation in food intake

In the wild, food availability can show considerable seasonal fluctuation resulting in associated variation in body weight and food intake for animals (Loudon, 1994). Seasonal rhythms in energy storage and expenditure appear to have evolved in a way for the animal to anticipate annual changes in the external environment thereby allowing an animal to adjust its physiology and behavior in preparation for the changing seasons (Ebling and Barrett, 2008). These adjustments are clearly important adaptations to environmental change and in the wild, appear to be driven by changes in external day length signals (Rousseau et al., 2003). However, these seasonal fluctuations in food intake and body weight are also observed in the presence of *ad-libitum* food and are exhibited under standard laboratory conditions (Mercer, 1998). As it is the case that when food is kept constant and feeding-related behaviors show changes that are linked with a seasonal pattern (Ferguson and Maier, 2013), it suggests that other, hard-wired physiological responses may mediate seasonal changes in behavior. We hypothesize that changes in behavior may be brought about by fluctuations in the level or neural responses to circulating hormones.

The definition of season for the purposes of this review paper will be based on photoperiod length. As such, short day (SD) will be synonymous with autumn and winter months when daylight is typically less than 12 h. Long day (LD) will be used interchangeably with the summer months when daylight is typically more than 12 h. The general guiding behavioral principle is that during LD photoperiods, food intake is increased to accumulate fat and during SD photoperiods, there is a decrease in food intake and a utilization of the excess fat (Schuhler and Ebling, 2006). As an example, Otsuka and colleagues found that during SD conditions (winter), rats have a lower body weight and epididymis fat mass compared to rats exposed to LD (summer) conditions (Otsuka et al., 2012). We hypothesize that seasonal changes in the sensitivity to and circulating levels of glucocorticoids and the feeding-related hormones ghrelin and leptin may be contributing factors to these seasonal variations in food intake. Indeed, non-hibernating species [e.g., sand rats (*Psammomys obesus*), Fischer 344 rats, golden hamsters (*Mesocricetus auratus*)] have been found to have higher circulating blood levels of glucocorticoids in the winter months than in the summer (Gutzler et al., 2009). Evidence also suggests that the sensitivity to and secretion of ghrelin and leptin are seasonally dependent, with relationships that are subject to photoperiodic regulation. In this respect, ghrelin concentrations decrease in the winter (discussed further below) in association with reduction in food intake showing seasonal variation in the level of circulating ghrelin (Bradley et al., 2010). As well, central or peripheral ghrelin administration increases food intake 2-fold after administration during summer months; an effect that is absent in the winter months showing changes in ghrelin sensitivity (Bradley et al., 2010). With respect to leptin, increased food intake and weight gain in LD are associated with high circulating leptin and decreased intake and weight loss in SD are associated with low circulating levels of leptin, showing changes in circulating levels (Rousseau et al., 2003). Seasonal leptin resistance has also been found showing higher sensitivity to leptin in the winter, thereby decreasing food intake, and lower sensitivity in the summer (Rousseau et al., 2003). Therefore, this review will examine the seasonal regulation of both stress- and feeding-related hormones as contributing factors in seasonal variation in feeding-related behaviors.

## Glucocorticoids

A stressor can be defined as a situation or event that an organism recognizes as threatening or challenging. Stressors can be either acute or chronic. An acute stress can be defined as a single exposure to a threatening situation (e.g., inescapable tailshock; Frank et al., 2013). Forms of chronic stress occur when an organism is exposed to a number of unpredictable stressors (e.g., restraint, water deprivation, food deprivation, circadian disruption) over an extended period of time [e.g., 1–2 weeks or longer (Frank et al., 2013)]. Both acute and chronic stressors elicit a series of physiological and behavioral reactions that mobilize stored energy and facilitate an organism's ability to cope (Anisman and Merali, 1999). The two most commonly studied physiological systems that respond to stress are the sympathetic nervous system and the neuroendocrine system (McEwen and Sapolsky, 1995; McEwen, 2000). The neuroendocrine system response to stress begins with an activation of the hypothalamic-pituitary-adrenal (HPA) axis. The hypothalamus secretes corticotrophin-releasing hormone (CRH) and arginine vasopressin (AVP) to affect the anterior lobe of the pituitary gland. When CRH and AVP bind to the anterior pituitary, the secretion of adrenocorticotropic hormone (ACTH) ensues. This hormone travels through the bloodstream to the adrenal glands, which respond by releasing glucocorticoid hormones into general circulation. Glucocorticoids bind to receptors on cells throughout the body, triggering a number of metabolic reactions that help direct oxygen and nutrients to the stressed body site (Harbuz and Lightman, 1992; Anisman and Merali, 1999). Glucocorticoid binding in the brain modulates a variety of processes, including attention, feeding, mood, and memory (Fietta and Delsante, 2009). Because a stressful event usually elicits glucocorticoid secretion, glucocorticoid levels are often used as a relative index of HPA axis activity (Johnstone et al., 2012) and hence, an approximation of the stress response.

Stress is known to alter feeding-related behaviors with both increases and decreases being observed. Stress-induced changes in feeding responses are due to a number of contributing factors, including the level of circulating glucocorticoids (for review see Maniam and Morris, 2012). With this in mind, one possible reason for changes in food seeking behaviors in the winter may be due to either basal or stress-induced changes in circulating glucocorticoids.

### Seasonal variation in glucocorticoids

Many organisms have internal mechanisms that allow them to anticipate changing seasons (circannual rhythms) in order to prepare for migration, changes in food availability or periods of reproduction (Gwinner, 2003; Paul et al., 2008). Many species also show circadian rhythms that are typically synchronized to the environment by regular cues such as light and temperature (Dickmeis et al., 2013). The interaction between these two types of rhythms is not clear but it is interesting to speculate that circadian fluctuations in circulating levels of glucocorticoids may be an internal mechanism used to anticipate the changing of the seasons based on photoperiod. In addition to its stress reactivity, circulating levels of glucocorticoids show daily variation whereby levels peak prior to the active period (day for diurnal species and night for nocturnal species; Chung et al., 2011; Ota et al., 2012). As the photoperiod changes from LD to SD conditions, it is possible that the circadian fluctuation of circulating glucocorticoid levels will adjust in terms of overall plasma concentration and possibly, time of peak secretion (Matchock et al., 2007). A study by Vondrasova et al. (1997) looked at the LD effects of summer on human glucocorticoid rhythms and the diurnal fluctuations. They found that glucocorticoids rose earlier in the day during the summer than the winter months (Vondrasova et al., 1997). This may be a hard-wired, internal signaling mechanism for the organism to anticipate changes in food availability and start changing its feeding patterns. Once the SD period sets in, the question remains as to whether there are elevations in basal levels of glucocorticoids and whether these elevations in glucocorticoids (either basal or those brought on by a stressor) would serve to alter SD feeding patterns.

#### Animal studies

Changes in the levels of circulating glucocorticoids are often used to assess HPA activation in response to stressful stimuli. There is evidence indicating that mammals, reptiles, amphibians, and birds display seasonal changes in both basal and stress-induced plasma glucocorticoid concentrations that may alter behavioral output (Romero, 2002; Romero et al., 2008). Studies on non-hibernating mammals [e.g., sand rat, adult male white-footed mouse (*Peromyscus leucopus*), subterranean rodent (*Ctenomys talarum*), bison bull (*Bison bison*), Belding's ground squirrel (*Spermophilus beldingi*)] in both the wild and in the laboratory have shown marked seasonal changes in both circulating basal levels of glucocorticoids and stress-induced changes in circulating levels of glucocorticoids (Amirat et al., 1980; Amirat and Brudieux, 1984, 1993; Mooring et al., 2006; Nunes et al., 2006; Pyter et al., 2007; Vera et al., 2011). Overall, these studies indicate that non-hibernating species have increased basal and stress-induced glucocorticoid levels in the fall and winter months compared to the spring and summer months (Amirat et al., 1980; Amirat and Brudieux, 1984, 1993).

Specifically, when blood samples were taken from the desert sand rat, basal levels of glucocorticoids were low during early June, increased during autumn and peaked to their highest levels during the winter months (Amirat et al., 1980; Amirat and Brudieux, 1993). In studies using rats and mice where light and dark cycles were manipulated to mimic winter (SD) and summer (LD) months, basal glucocorticoid levels tended to be higher during the SD periods (winter) than LD periods (summer) (Ottenweller et al., 1987; Pyter et al., 2007; Otsuka et al., 2012). When the photoperiod was manipulated in Syrian hamsters, they showed modification of plasma concentrations of glucocorticoids such that the peak level of circulating glucocorticoids during SD was lower compared to LD (Ottenweller et al., 1987).

In mice, SD periods are associated with an increased HPA-axis responsiveness to stressful stimuli (Pyter et al., 2007; Otsuka et al., 2012). SD photoperiods have also been shown to be associated with an increase in stress-induced circulating glucocorticoid levels in response to restraint stress (Pyter et al., 2007). Changes in glucocorticoid levels in response to exogenous administration of dexamethasone have also shown seasonal variations. After dexamethasone administration, plasma glucocorticoid levels showed a large, rapid increase that persisted for a longer timeframe during the winter (February) than the summer (June) (Amirat et al., 1980; Amirat and Brudieux, 1984, 1993). The work with animals all points to the tentative conclusion that circulating glucocorticoid levels—both basal and stress-induced—are higher during the winter months than in the summer months.

#### Human studies

Seasonal variation in basal glucocorticoid levels has also been noted in humans and has been examined in healthy participants using salivary swabs, urine collection, or blood plasma levels (Walker et al., 1997; King et al., 2000; Thorn et al., 2011). Overall, there is some consensus that basal levels of circulating glucocorticoids are lower during the spring and summer and peak during the fall and winter (Walker et al., 1997; King et al., 2000). Concentrations of urinary glucocorticoid levels were reported to be higher during December and January compared to the rest of the year (Hansen et al., 2001). A study by Vongrasova and colleagues manipulated the photoperiod in the summer (Vondrasova-Jelinkova et al., 1999). They found that by shortening the photoperiod in the summer to mimic day length as would occur in winter, they could match the elevated glucocorticoid levels as those observed during the winter. Due to the circadian nature of fluctuating glucocorticoid levels throughout the day, consistency in sample collection is an important factor in human studies. For example, the study by King and colleagues showed a significant effect of seasonality when they gathered saliva samples during both the morning and evening finding that basal levels of glucocorticoids were lowest in the spring and highest in the winter and fall (King et al., 2000). Therefore, there appear to be consistent findings of seasonal variation in human basal glucocorticoid levels with higher levels in SD conditions.

While there is a dearth of studies examining seasonal variation in stress-induced changes in glucocorticoid levels in humans, the available literature suggests more pronounced stress-reactivity in the winter than in the summer. In an examination of seasonal variation in stress-induced changes in circulating levels of glucocorticoids, hourly blood samples were obtained from medical students under the influence of a stressful event (an exam; Malarkey et al., 1995). These students reported that the examinations did result in a significant elevation in psychological stress. In addition, there was a significant effect of examination stress on the increase in mean daytime ACTH levels during autumn, but not during the spring. In a similar study, an academic stressor was found to increase circulating levels of glucocorticoids more so in the fall-winter months than in the summer months (Matalka and Sidki, 1998).

### Glucocorticoids and feeding-related behavior

Circulating levels of glucocorticoids, either basal or stress-induced changes, have been shown to influence food choice, body weight and appetite in both humans and animals (Oliver and Wardle, 1999; Baran et al., 2009; Liu et al., 2011; Schwabe and Wolf, 2011) and this may be an important contributing factor to seasonal variation in food intake.

#### Animal studies

Animal studies in the laboratory have revealed that, overall, stress-induced changes in circulating glucocorticoids or exogenous administration of glucocorticoids (thereby elevating basal levels) leads to a decrease in food intake (Levine and Morley, 1981; Morley et al., 1983; Liu et al., 2011). In laboratory studies under free-feeding conditions, when rats were administered exogenous glucocorticoids over a period of days, mimicking a chronic elevation in basal levels of glucocorticoids as would be observed in SD, winter months, a decrease in food intake leading to a decrease in body weight was reported (De Vos et al., 1995; Liu et al., 2011). A study by Liu et al. (2011) administered a high dose (15 mg/kg/day) and a low dose (5 mg/kg/day) of hydrocortisone to rats to study food intake and body weight change (Liu et al., 2011). They found that after chronic administration of the high dose of hydrocortisone (similar to elevated basal levels of circulating glucocorticoids as observed in SD, winter, conditions), food intake was reduced [see also De Vos et al. (1995), Konno et al. (2008) for similar results of decreased food consumption after dexamethasone treatment]. Because chronic hydrocortisone treatment mimics high levels of basal circulating glucocorticoids, as would be observed in SD conditions, results indicate that elevated circulating levels of basal glucocorticoids, rather than food scarcity (as occurs in SD conditions) may be a causal, contributing factor to the decreased food intake in laboratory rats.

Chronic and acute stressors appear to affect food intake differently. Chronic stressors tend to decrease food intake (Levine and Morley, 1981; Liu et al., 2011; Nyuyki et al., 2012), whereas acute stressors often lead to an increase in food intake (Levine and Morley, 1981; Morley et al., 1983). However, the severity of the stressor—either chronic or acute—may also be a critical factor in the modulation of food intake. For example, a sudden drop in food intake is likely the result of a severe, acute stressor in rats (Armario, 2006). A study by Levine and Morley (1981) used tail pinch as a mild, acute stressor to investigate food intake in rats in the laboratory. During the acute tail pinch procedure, rats ate steadily over a 5-minute period with increased eating being observed during a second trial, 15 min after the first. The chronic tail pinch group consumed less food than the acute group and exhibited leading to a decrease in body weight compared to controls (Levine and Morley, 1981). Another type of chronic stressor is chronic subordinate colony housing where four male rats are housed together with a larger male rat. This chronic stressor has been shown to decrease body weight gain and increase plasma glucocorticoids when compared to controls (Nyuyki et al., 2012). Although chronic stress appears to decrease food intake when only normal rat chow is present, palatable food (e.g., high fat, sucrose) consumption may increase in response to a chronic stressor (Dallman et al., 2003). Under chronic restraint stress conditions, when rats were given a choice between chow or dense lard and sucrose food (a palatable food), the rats undergoing chronic stress were more likely to choose and eat the palatable food but decrease intake of the regular chow (Pecoraro et al., 2004).

#### Human studies

In humans, stress is associated with both increases and decreases in food intake (Oliver and Wardle, 1999; Epel et al., 2001). The feeding response to stress can vary depending on individual differences, type of food available, and the type of stressor (chronic, acute, severe, moderate; Maniam and Morris, 2012). For example, acute stress affects food intake in humans subjects differently depending on the general state of chronic stress in their lives (Rutters et al., 2009; Tryon et al., 2013). Stress-induced changes in glucocorticoid levels are individualized as some individuals show a larger glucocorticoid response following a stressor than others (Epel et al., 2001; Rutters et al., 2009) and this in turn can affect feeding behavior. In humans, food intake is more likely to increase during a stressful event if a palatable food is readily available (Oliver and Wardle, 1999; Dallman et al., 2003, 2005; Zellner et al., 2007; Bennett et al., 2013). In a study by Oliver and Wardle (1999), an approximately equal number of participants reported eating more as eating less when stressed; however, the majority of participants reported an increase in snacking under stressful situations (Oliver and Wardle, 1999). It may be the case that stress alters food choice, with participants choosing more sweet snacks such as chocolate and cake rather than savory, meal-type foods (Oliver and Wardle, 1999; Dallman et al., 2003; Bennett et al., 2013). Gender may also play a role in food choice (Zellner et al., 2006, 2007). A study by Zellner et al. (2007) used unsolvable anagrams to stress participants. They placed four bowls of snack foods in the room; two healthy, two unhealthy. The men in the no-stress group ate more of the unhealthy foods (such as chocolate) than the men in the stress group (Zellner et al., 2007). Conversely, in a study investigating stress in women, it was found that under stress conditions, women were more likely to increase consumption of the unhealthy foods (Zellner et al., 2006).

### Glucocorticoid summary

Overall, while the animal data indicate that chronic or severe stress-induced elevations in circulating levels of glucocorticoids may reduce food intake, the picture is much more complicated in humans. However, the converging lines of evidence showing (1) reduced food intake in SD conditions, (2) elevated basal levels of circulating glucocorticoids in SD conditions, and (3) experimental findings showing stress-induced elevations in glucocorticoid levels and changes (reductions in animal/rodent models) in food intake point to the possibility that seasonal variation in food intake may be due, in part, to changes in circulating glucocorticoid levels. Elevations in basal levels of glucocorticoids, as happen during SD photoperiods, may be a factor in not simply reducing food intake, but, rather, changing food selection processes. If particular foods are not present, the organism may not be inclined to engage in feeding behavior and fat reserves may be depleted. On the other hand, if particular foods are present during the winter months, consumption of those foods may be elevated.

## Ghrelin

While it seems that elevated levels of basal or stress-induced glucocorticoids during the winter months may be a factor in altering food intake, the contribution of hormones involved in feeding responses may show more robust seasonal fluctuations. In this case, levels of hormones that stimulate feeding would be predicted to be lower in the winter than in the summer. One such hormone is ghrelin.

Ghrelin, a 28- amino acid peptide, was the first identified circulating hormone that, when elevated either exogenously or endogenously, promotes feeding (Kojima et al., 1999). Ghrelin is synthesized primarily within the gastric oxyntic mucosa of the stomach and is an endogenous ligand for the growth hormone secretagogue G-protein coupled receptor (GHS-R). Within the central nervous system, GHS-R are expressed in the hypothalamus, the ventral tegmental area (VTA), the dorsal (DRN) and medial (MRN) raphe nuclei and the hippocampus (Zigman et al., 2006). The high expression of GHS-R in these regions may explain the role of ghrelin in meal initiation and weight regulation. These brain areas are involved in a number of homeostatic processes that underlie a variety of regulatory behaviors, such as feeding (Gil-Campos et al., 2006).

Ghrelin is released in response to acute and chronic changes in nutritional state and, while the factors involved in the regulation of ghrelin secretion are still under investigation, blood glucose levels appear to contribute to the rise and fall of ghrelin levels (Shiiya et al., 2002). Endogenous ghrelin levels increase prior to feeding when an animal is hungry and decrease after feeding (Tschop et al., 2000; Theander-Carrillo et al., 2006). Decreased levels of ghrelin have been shown to decrease the motivation of rats to obtain reward (Skibicka and Dickson, 2011). Exogenous application of ghrelin has been shown to dose-dependently increase food intake 24 h after a single injection in the third ventricle, the DRN or the hippocampus (Carlini et al., 2002, 2004). Carlini et al. (2004) also looked at how soon after a ghrelin injection into the DRN or hippocampus food intake increased. They found that the increase in food intake after ghrelin injections could be seen as early as 1 h after injection. They also saw that at the highest dose (3.0 nmol/ul), food intake was double that of control animals (Carlini et al., 2002, 2004). Abizaid et al. (2006) used a repeated-fast protocol in wild type, GHS-R knock-out and ghrelin deficient mice. The repeated-fast procedure consisted of two phases. During the first phase, food was removed from the cage overnight then returned in the morning for 6 h. Food intake was measured once the food was replaced at various time points. For the second phase, food was removed after the sixth hour until the next morning when food was again re-placed for another 6 h. They found that both the ghrelin and GHS-R deficient groups showed lower food intake compared to the wild type group. These studies delineate an important role of ghrelin in stimulating appetite and increasing feeding behaviors as well as increasing the motivation to seek out food (for review see Hosoda et al., 2002; Kojima and Kangawa, 2002; Wells, 2009).

### Seasonal variation in ghrelin

The mechanisms controlling the ability of animals to prepare for and anticipate seasonal changes seems to be hardwired—consisting of both hormonal and neuronal interactions that influence body mass, food intake and metabolism (Underwood, 1971; Haga, 1993; Korhonen and Saarela, 2005). Animals that live in climates with predictable seasonal changes in food availability must acquire metabolic strategies that compensate for these seasonal changes (Bradley et al., 2010). Animals typically increase food intake during the summer in an attempt to accumulate fat stores that can then be utilized in the winter when food is scarce (Fuglei et al., 2004; Bradley et al., 2010; Janzen et al., 2012). Ghrelin has been identified as a potential candidate for mediating seasonal changes in metabolism, behavior, and food intake. However, there is conflicting research as to the effects of seasonal variation in the responsiveness and expression of ghrelin, which appears to stem from species-specific effects of the hormone. Here we will focus on the effect of ghrelin in mammals.

In mammals, the seasonal effects of ghrelin are dependent on (1) the level of circulating ghrelin that is produced and (2) the neural and behavioral responsiveness to ghrelin. The Arctic fox displays seasonal fluctuations in circulating ghrelin levels in relation to seasonal adjustments in fat deposition, metabolic rate and food-related behaviors (Fuglei et al., 2004). In this species, plasma levels of ghrelin are lowest during winter when food intake is suppressed and highest in the summer when food is more abundant (Fuglei et al., 2004; Mustonen et al., 2005a). The low levels of circulating ghrelin that occur in the winter months may stimulate the utilization of fat stores as an adaption to food restriction as evidenced by a negative correlation between ghrelin levels and free fatty acids (FFA; Fuglei et al., 2004).

The finding of increased utilization of fat stores is supported by findings that there are large seasonal variations in body fat and weight in the Arctic fox. In summer months when food levels are highest, the deposition of fat takes place, as approximately 20% of their total body weight is fat. By the onset of winter, this accumulation of fat is rapidly mobilized and, as a result, fat levels are at their lowest by the winter's end (Fuglei et al., 2004). Interestingly, circulating ghrelin fluctuates from intermediate to high levels during autumn when voluntary food intake is shown to be highest and the animals are accumulating fat. These increased ghrelin levels may underlie the Arctic foxes ability to gather adequate energy stores for the winter season by increased food intake that increases fat stores in the autumn, a process known as wintering (Mustonen et al., 2005a).

In the Siberian hamster, ghrelin is a potential component of the neurohormonal mechanism that regulates seasonal changes in food intake. In this species, changes in day length have been shown to initiate changes in metabolism, behavior, body mass and food intake (Steinlechner and Heldmaier, 1982). During SD periods, these animals reduce the cost of thermoregulation by reducing food intake and body mass (Tups et al., 2004; Bradley et al., 2010). This appears to be achieved by a change in the responsiveness to ghrelin rather than a reduction in the level of circulating ghrelin as a change in day length did not alter fasting levels of ghrelin (Tups et al., 2004). Thus, during SD, body mass is reduced in part because of a decreased responsiveness to ghrelin which in turn attenuates feeding (Keen-Rhinehart and Bartness, 2005; Bradley et al., 2010). However, during LD periods (summer), there is an attempt to increase fat stores for winter survival. As such, the responsiveness to ghrelin increases leading to an increase in the motivation to seek food and feed (Bradley et al., 2010). Bradley et al. (2010) found that Siberian hamsters ate more during the LD than the SD following i.p. ghrelin injections (same dose). Although day length does not affect the level of circulating ghrelin in these animals, after fasting, hamsters in LD conditions ate more than hamsters in SD conditions (Bartness and Wade, 1985a; Tups et al., 2004) further suggesting a reduced responsiveness to ghrelin signaling. Possibly, a lower threshold for behavioral responsiveness to ghrelin exists which may lead to more frequent meals in LD conditions and increased motivation to feed in an attempt to increase fat stores for winter survival (Bradley et al., 2010).

### Ghrelin and feeding behavior

Various studies have indicated that ghrelin has a powerful impact on a number of aspects of feeding-related behaviors. Operant conditioning has been used to assess the motivational properties of rewarding food by assessing the magnitude of acquired behaviors (i.e., nose pokes and bar presses) that are directed toward obtaining the food reward (Thorndike, 1911; Hodos, 1961; Hodos and Kalman, 1963). Numerous studies have shown that ghrelin increases operant lever pressing for various food rewards including high fat diet (HFD), sucrose, peanut butter and chocolate pellets in both mice and rats (Perello et al., 2010; Skibicka et al., 2011). Further suggestive of an increased motivational state to obtain food reward, rats or mice that were given intra-VTA ghrelin infusions earned more food rewards during a progressive ratio task (Perello et al., 2010; Skibicka et al., 2011). This effect was matched by the finding that rats given ghrelin injections increased their chow intake when food was presented after conditioning while rats (hungry or satiated) injected with the GHS-R antagonist showed reduced operant responding (Skibicka et al., 2011).

Another behavioral task that has been used to highlight the contribution of ghrelin to feeding-related behaviors is the conditioned place preference (CPP) task. Using the CPP task, Disse et al. (2010) conducted a study using both wild type and GHS-R knock-out mice. They first looked at the effects of natural increases in ghrelin as would be brought about by food restriction (mice were restricted to 50% of their normal food intake during a 24 h period). They found that a short exposure to the food-paired side during training elicited a strong CPP in WT mice as measured by increased time spent in the food-paired side. This effect was absent in GHS-R knock-out mice, indicating that GHS-R KO reduced the facilitation of learning the CPP with short training exposures brought about by food restriction (Disse et al., 2010). The next part of the study investigated the effect of exogenously administered ghrelin on the CPP task by administering ghrelin i.p. 20 min before the conditioning sessions. It was found that pretreatment with ghrelin increased the amount of time spent in the food-paired side over and above the time displayed by the vehicle-treated group (Disse et al., 2010). Furthermore, by administering a GHS-R antagonist to rats prior to the conditioning sessions, (Egecioglu et al., 2010) found that the ability of a rewarding food to elicit a CPP was suppressed. These findings highlight the conclusion that ghrelin signaling may serve a critical role in the pathways involved in the motivation required for reward-related behaviors.

#### Ghrelin and dopamine

The mechanisms through which ghrelin promotes food intake and body weight regulation are multifaceted, stimulating not only food intake, but also enhancing the rewarding properties of food and the motivation to acquire food (for reviews see Skibicka and Dickson, 2011; Perello and Zigman, 2012). Feeding-associated reward is governed by the mesolimbic dopamine (DA) pathway that consists of cell bodies located in the VTA sending projections to multiple nodes in the Acb, amygdala, prefrontal cortex, and hippocampus (Nestler and Carlezon, 2006). These projections are associated with food reward and food seeking behavior (Richardson and Gratton, 1998; Bassareo and Di Chiara, 1999). In addition to DA projections in the mesolimbic pathway being activated by the ingestion of rewarding and palatable food, these projections are activated by ghrelin (Abizaid, 2009) as GHS-Rs are present on approximately 60% of VTA DA neurons (Abizaid et al., 2006; Jerlhag et al., 2006; Zigman et al., 2006).

Various experiments have investigated the influence of ghrelin on the DA-containing pathways in relation to reward-related behaviors. For example, Jerlhag et al. (2006, 2010, 2011b) measured locomotor activity after ghrelin injections into the third ventricle. They found that after ghrelin injections, locomotor activity increased; an indication of increased DA output. Following direct injections of ghrelin into the VTA or the third ventricle, an increase in the frequency of action potentials in VTA DA neurons was noted as well as increased DA turnover into the Acb (Abizaid et al., 2006; Jerlhag et al., 2006, 2010). In addition, increased feeding was observed when ghrelin was infused into the VTA, suggesting that ghrelin may modulate DA activity in the VTA reward pathway to enhance feeding responses (Abizaid et al., 2006).

### Ghrelin summary

The regulation of ghrelin secretion appears to depend on nutritional state potentially arising from blood glucose levels; when an organism is hungry, and thereby low levels of blood glucose, circulating levels of ghrelin would rise and stimulate feeding behaviors via activation of receptors in the brain. In mammals, the seasonal effects of ghrelin are dependent on (1) the level of circulating ghrelin that is produced and (2) the neural and behavioral responsiveness to ghrelin. Circulating ghrelin levels are decreased in SD conditions and ghrelin reactivity is also decreased in the winter. Both of these physiological responses might ultimately impede DA function (neural activation and output) and impede food-seeking and food intake in SD conditions.

## Leptin

Leptin is a peptide hormone that was first described in 1994 (Casanueva and Dieguez, 1999). Leptin is the product of the obese gene and is expressed and secreted exclusively by adipocytes in the periphery (Zhang et al., 1994). Leptin affects areas of the central nervous system involved in the regulation of energy balance with prompt feedback regarding the status of the bodies energy stores and energy flux to help modulate food intake and energy homeostasis (Zhang et al., 1994; Ahima and Flier, 2000; Perry et al., 2010). Leptin functions as an afferent signal in a negative feedback loop that controls adipose tissue mass and reduces food intake. The amount of leptin produced by adipocytes is proportional to body fat mass. Leptin is shown to decrease food intake, as exogenous leptin administration decreased food intake and body weight (Ahima and Flier, 2000; Friedman, 2004; Domingos et al., 2011). Leptin is thought to be another major factor in energy balance and is hypothesized as having the opposite effect as ghrelin on food intake and body weight (Tups et al., 2004; Schmid et al., 2005). A negative correlation between circulating ghrelin and leptin exists, as leptin appears to inhibit food intake and ghrelin has been shown to increase food intake. Thus, when leptin is increased, ghrelin is reduced and food intake is attenuated (Tups et al., 2004; Schmid et al., 2005). Barazzoni et al. (2003) showed that even under caloric restriction, which would naturally increase circulating ghrelin levels, subcutaneous leptin infusions in lean rats prevented this natural rise in serum ghrelin levels. This finding of reduced serum ghrelin when leptin is injected during food deprivation highlights the role of leptin in the negative feedback loop that controls body weight and food intake.

Leptin is released in response to acute and chronic changes in nutritional state. Endogenous leptin levels are low prior to feeding when an animal is hungry and will increase as an animal becomes satiated (Tschop et al., 2000; Davis et al., 2011). Leptin appears to increase energy expenditure and decrease food intake which results in a decreased body weight and body fat mass (Campfield et al., 1995; Dileone, 2009; van Zessen et al., 2012). The role of leptin in maintaining energy homeostasis has also been shown to regulate reward- related behaviors (Davis et al., 2011). As leptin and its receptors have been found in the VTA, it is not surprising that leptin has been shown to regulate effort-based responding for food reward (Davis et al., 2011). Leptin is thought to accomplish this by decreasing DA release and by opposing the actions of ghrelin in the VTA (Davis et al., 2011). Viral knock-down of the leptin receptors in the VTA not only increased food intake of balanced or high fat diet, but also elevated the preference for sucrose or high fat diets (Hommel et al., 2006; van Zessen et al., 2012). It seems that in addition to being involved in the hypothalamic control of energy expenditures, body mass, and food intake, leptin is also involved in the mesolimbic dopaminergic system control over reward-based feeding and responding (Caro et al., 1996; Davis et al., 2011). The function of leptin to signal the central nervous system about the state of energy stores is extremely important as adequate reserves are important for survival (Mustonen et al., 2005a; Schmid et al., 2005).

### Seasonal variation in leptin

Animals have developed both physiological and behavioral strategies to anticipate and overcome the different challenges that accompany the changing of seasons (Zhao and Wang, 2006). Photoperiod length differs across seasons, as winter has reduced periods of sunlight compared to summer. Photoperiod plays an important role in mediating the seasonal variation in body mass and energy homeostasis (Bartness and Wade, 1985b; Bartness and Goldman, 1989; Zhao and Wang, 2006). Similar to ghrelin, leptin fluctuates in accordance with the seasonal fluctuations in energy intake, body mass and fat mass. Seasonal changes in energy intake, body mass, body fat, and serum leptin levels occur independently from food availability, which suggests a hard-wired, evolutionarily conserved mechanism (Klingenspor et al., 1996, 2000; Zhao and Wang, 2006). It seems possible that during different seasons, leptin is utilized differently from those signals induced by negative energy balance, created by controlled food deprivation (Adam and Mercer, 2001). Here we will focus on the effect of leptin in seasonal non-hibernating mammals and how changes in photoperiod affect the responsiveness of leptin and the impact on energy intake and body fat mass.

In seasonal mammals, such as the Siberian hamster, weight loss resulting from the winter months is associated with low circulating levels of leptin compared to the summer months (Adam and Mercer, 2001). There also appear to be changes in the sensitivity to leptin feedback as expression of the leptin receptor gene in the hypothalamus is lower during the winter months whereas, under natural conditions of food deprivation, expression of the gene is increased (Adam and Mercer, 2001). The changes in leptin responsiveness or sensitivity and concentration during seasonal variation have been studied in a number of seasonal mammals such as Siberian hamsters, Arctic foxes, and Brandt's voles; all discussed here.

In the Siberian hamster, the effects and responsiveness of leptin during different seasons has been investigated via manipulating the photoperiod and infusing leptin at different time points. Under normal conditions, there is a robust positive correlation between total body fat mass and plasma leptin concentration (Korhonen et al., 2008). There is significant body weight loss, in terms of adipose tissue amount, during the transition from the obese LD phenotype to the leaner SD phenotype (Klingenspor et al., 1996). The mobilization of adipose tissue during the SD period is associated with decreased leptin plasma concentration. For example, leptin levels are 2–3 times higher in Siberian hamsters during the LD as opposed to the SD condition (Klingenspor et al., 1996; Tups et al., 2004). The appetite-depressing effects of leptin are greater in Siberian hamsters exposed to SD conditions than those in LD conditions suggesting that SD-exposed hamsters have increased sensitivity to leptin (Klingenspor et al., 2000). Likewise, Rousseau et al. (2002) demonstrated that, when all other physiological parameters were controlled, chronic leptin infusions only resulted in body weight and fat loss in Siberian hamsters exposed to a SD conditions [similar findings were reported by Atcha et al. (2000)]. Thus, it appears that photoperiod may alter the sensitivity to leptin-induced seasonal changes in body weight, food intake, and fat mass in the Siberian hamster.

Brandt voles also have an increased energy mobilization in SD periods compared to LD. Compared to LD voles, voles acclimated to SD conditions have decreased levels of serum leptin, decreased body fat mass and increased energy intake (Zhao and Wang, 2006). These variations in leptin serum levels, body composition, and energy intake between SD and LD were found to be independent from both temperature and food availability, suggesting that photoperiod length is a critical factor in this effect (Zhao and Wang, 2006). The decreased body weight and body mass in winter displayed by Brandt voles indicate that these animals have evolved a physiological adaption to maintain a low body mass in winter months (Flier, 1998; Li and Wang, 2005; Zhao et al., 2010). Additionally, the decrease in leptin plasma concentration during the SD photoperiod suggests that leptin may act as a starvation signal to increase energy intake to help cope with the conditions during SD photoperiods (Li and Wang, 2005).

The Arctic fox also expresses seasonal changes in leptin sensitivity (Mustonen et al., 2005a). The Arctic fox exhibits a pattern of seasonal fattening with fat accumulation starting in August and September and peaking in November and December (Fuglei et al., 2004). In the autumn months, voluntary food intake is increased, which leads to an accumulation of fat to prepare for the reduced food availability in winter (Fuglei et al., 2004; Mustonen et al., 2005a). The increased fat storage observed in autumn is accompanied by increased circulating plasma leptin concentrations (Mustonen et al., 2005a). This increase in plasma leptin concentrations does not seem to induce the normal loss of appetite, body weight and fat storage during autumnal fatting that is normally associated with leptin (Mustonen et al., 2005a). It seems that leptin does not act as an anorectic hormone during the autumnal fatting, as it is important for the Arctic fox to prepare for the harsh winter by maximizing feeding, with the goal of increasing body fat (Mustonen et al., 2005a). This decreased leptin sensitivity during autumn is not present in winter, as the gradual reduction in body mass and fat stores are accompanied by decreased leptin levels. This suggests that in the Artic fox, leptin functions as an indicator of adiposity during the winter fat mobilization period (Mustonen et al., 2005a).

The seasonal variation in leptin seems to be an evolutionary mechanism to anticipate changing environments. Animals that live in climates with predictable seasonal changes in food availability must acquire metabolic strategies that change according to the season (Bradley et al., 2010). It seems that leptin may be an integral component of the physiological processes needed to regulate appetite in anticipation of changing seasons. However, as is seen in the Artic fox during autumn, there are other times when animals may be insensitive to changes in blood leptin levels (Mustonen et al., 2005a). Another possibility is that leptin does not function as an acute indicator of body adiposity in seasonal animals, but rather, as a signal of nutritional status. This is evidenced by the positive correlation between plasma leptin concentrations and body composition in the Arctic fox, Siberian hamster, and Brandt voles when all circannual data are analyzed together (Mustonen et al., 2005a).

### Leptin and feeding behavior

The mechanisms for the ability of leptin to inhibit food intake and regulate body weight/adiposity composition are multifaceted. Leptin not only inhibits food intake but has also been shown to reduce both the rewarding properties of food and the motivation to acquire food (Davis et al., 2011). As with ghrelin, both operant conditioning and the CPP have been used to assess the role of leptin in the motivational properties of food. Injections of leptin have been shown to decrease progressive ratio responding for HFD and sucrose, as well as impair the acquisition and expression of a CPP for HFD (Figlewicz, 2003; Figlewicz et al., 2006; Hommel et al., 2006; Davis et al., 2011). These data suggest that motivation to acquire food is decreased by leptin. The ability of leptin to decrease the motivation to obtain food has been further studied by using knockdown of the leptin receptor (LepR) and LepR antagonists; both of which increase progressive ratio responding for a HFD (Davis et al., 2011). These findings suggest that leptin signaling and its receptors may play a critical role in the pathways involved in food-related behaviors.

#### Leptin and dopamine

Leptin can modify food-based behaviors by altering the function of the mesolimbic DA pathway (Figlewicz and Woods, 2000; Dileone, 2009; Perry et al., 2010). The rewarding aspects of feeding are governed by the mesolimbic DA pathways which are activated by the ingestion of rewarding and/or palatable food and are inhibited by leptin (Davis et al., 2011). LepR are expressed in the VTA and 75–90% of LepR-positive neurons are DA neurons (Hommel et al., 2006). Furthermore, direct application of leptin into the VTA has been shown to attenuate the activation of these DA neurons (Hommel et al., 2006).

Elevations in midbrain leptin are capable of modulating feeding behavior and DA responses (Krugel et al., 2003; Hommel et al., 2006). Injection of leptin into either the third ventricle or the VTA has been shown to decrease food consumption, whereas when the effect of leptin is decreased in the midbrain, feeding increases (Figlewicz et al., 2003; Krugel et al., 2003). In the VTA, both viral knock-down of LepR and administration of leptin receptor antagonists have been shown to increase both normal chow and HFD, as well as increase the preference for both sucrose and HFD (Hommel et al., 2006; Davis et al., 2011). These effects on feeding may be a result of leptin-induced changes in DA concentration and DA neuronal firing as leptin administration into the midbrain has been shown to attenuate the firing of DA neurons (Hommel et al., 2006; Davis et al., 2011). In addition, injections of leptin into the VTA reduce DA release into the Acb. This effect might result from a direct inhibitory effect of leptin on VTA DA neurons (Krugel et al., 2003; Hommel et al., 2006). Leptin's effects on DA firing and DA turnover into the Acb seem to directly oppose the actions of ghrelin (Dileone, 2009).

### Leptin summary

Leptin functions as an afferent signal that is released in response to acute and chronic changes in nutritional state. Endogenous leptin levels are low prior to feeding when an animal is hungry and increase as an animal becomes satiated. With respect to seasonal changes in leptin function, there appears to be seasonal leptin resistance whereby there is a higher sensitivity to leptin in the winter, thereby resulting in decreased food intake, and lower sensitivity in the summer, thereby resulting in elevated food intake. Leptin signaling in the DA system may be one mechanism through which the motivation to seek out and consume food is reduced as would happen during SD photoperiods. With leptin receptors showing increased sensitivity during the SD winter months, activity in the DA mesolimbic system would be significantly reduced thereby reducing food seeking behaviors.

## Synthesis of findings

We sought to investigate whether changes in circulating glucocorticoids or changes in the physiological effects of ghrelin and/or leptin during the winter would impede the propensity for an animal to engage in food-related behaviors such as seeking and consuming food. A review of the literature on seasonal variation in basal and stress-induced levels of circulating glucocorticoids and ghrelin and leptin levels and sensitivity reveals several plausible explanations for reduced food seeking and consuming behaviors during SD photoperiods.

### Glucocorticoid-mediated attenuation of food seeking behaviors in winter

Studies reviewed indicate that non-hibernating species have increased basal and stress-induced glucocorticoid levels in the fall and winter months compared to the spring and summer months (Amirat et al., 1980; Amirat and Brudieux, 1984, 1993). While some animal studies have revealed that stress-induced increases in circulating glucocorticoids leads to a decrease in food intake (Levine and Morley, 1981; Morley et al., 1983; Liu et al., 2011) others have reported that repeated bouts of stress (i.e., chronic forms of stress) leads to increased food consumption (Pecoraro et al., 2004; Lutter et al., 2008; Pankevich et al., 2010). These differences could be due to an acute/chronic stressor procedure whereby acute stress seems to decrease food consumption while chronic stress increases food consumption. Indeed, seasonal variation in glucocorticoid responses to chronic and acute stress also seems to vary (Vera et al., 2011).

Circulating levels of glucocorticoids are elevated in association with goal-directed, reward-related behaviors (Schwabe et al., 2011). As shown by others (Kerr et al., 1991; Arbel et al., 1994; Bodnoff et al., 1995), elevations in circulating glucocorticoid concentrations by systemic administration or glucocorticoid receptor agonists, impair learning and memory processes. The relationship between stress, DA, and food seeking behaviors may or may not support the idea that elevated stress can specifically impair DA-mediated increases in behavioral output. Stress-induced increases in levels of circulating glucocorticoids has been reported to increase DA concentrations in the Acb shell (Brake et al., 1999, 2000) particularly in conditions when the DA system is activated (Cador et al., 1993; Rouge-Pont et al., 1995; Marinelli and Piazza, 2002) as would be the case when animals are seeking food. An acute injection of dexamethasone (8 mg/kg but not 4 or 2 mg/kg) reduces locomotor activity in mice and acute or chronic dexamethasone treatment (mimicking increased levels of circulating glucocorticoids) reduces DA-dependent amphetamine-stimulated increases in locomotor activity (Wrobel et al., 2005). A report has also shown that repeated restraint stress enhances DA uptake into striatal synaptosomes within the Acb, characterized by increased transporter capacity, potentially diminishing DA function (Copeland et al., 2005). As well, stress has been shown to downregulate D1 receptor binding in the Acb shell and upregulate D1 receptor binding in the VTA (Czyrak et al., 2003). In this scenario, even with elevated DA being produced by elevated, stress-induced circulating glucocorticoids, DA function would be lessened by elevated re-uptake and downregulated postsynaptic actions. This would then attenuate food-seeking behavior as seen in SD photoperiods.

### Ghrelin-mediated attenuation of food seeking behaviors in winter

A second possibility is that seasonal fluctuations in ghrelin could decrease the propensity for the animals to engage in food-seeking behaviors via an interaction with DA. As reviewed, ghrelin levels are lower in the winter than in the summer and animals are more likely to eat in excess and in higher caloric amounts during the summer potentially in preparation for the winter (Fuglei et al., 2004; Keen-Rhinehart and Bartness, 2005; Mustonen et al., 2005a,b; Bradley et al., 2010). This suggests that food-seeking behaviors would be increased in the summer and decreased in the winter. Numerous studies have shown that ghrelin increases operant lever pressing for various food rewards including HFD, sucrose, peanut butter, and chocolate pellets in both mice and rats (Perello et al., 2010; Skibicka et al., 2011). In terms of the functional outcomes in response to decreased ghrelin activity, ghrelin receptor antagonist injections have been shown to reduce operant responding for a sucrose solution (Landgren et al., 2011). Therefore, in the winter when ghrelin levels are low, a reduction in food-seeking, and potentially, consuming, behaviors would be predicted. Conversely, in the summer, these behaviors might be increased due to the elevations in ghrelin activity.

It is hypothesized that the actions of ghrelin on food-seeking behavior are due to interactions with the DA system. The ability of ghrelin to potentiate the DA reward system was studied by Abizaid et al. (2006) who found that ghrelin increased the frequency of action potentials of DA neurons in the VTA and increased DA turnover into the Acb. The ability of ghrelin to stimulate action potential firing in VTA DA neurons requires glutamatergic excitatory inputs (Andrews, 2011). As such, blockade of glutamatergic signaling following intra-VTA administration of the NMDAr antagonist AP5, blocked ghrelin-induced DA output in the Acb along with ghrelin-induced locomotor elevation (Jerlhag et al., 2011a). In this scenario, with low levels of ghrelin in the winter, the VTA-Acb circuit would show overall less responsivity to reward-related signals and reduce the “higher-order” aspects of food acquisition, such as motivation and cue responses (Narayanan et al., 2010). This would set up a condition whereby the animals would be less likely to engage in food-seeking behaviors.

### Leptin-mediated attenuation of food seeking behaviors in winter

A third possibility is that seasonal variations in changes in the responsiveness to leptin during the winter months would contribute to reduced food-seeking behaviors and reduced food intake possibly, again, via interactions with the DA system. Increased food intake and weight gain in LD photoperiods are associated with a relative insensitivity to high circulating leptin serum levels while decreased intake and weight loss in SD photoperiods appear to be an outcome of low circulating leptin but high sensitivity (Adam and Mercer, 2004). This heightened sensitivity to leptin during the winter SDs would lead to a decreased propensity to engage in food-seeking behaviors either through reductions in DA output or increases in anxiety-like behaviors.

Leptin-receptor expressing neurons are located in a subset of DA neurons in the VTA as well as the substantia nigra (Figlewicz, 2003). Leptin signaling in the VTA may be one mechanism through which the motivation to seek out and consume food is reduced (Hommel et al., 2006) as would happen during SD photoperiods. Viral-mediated reduction in the leptin receptor on these midbrain DA neurons can prolong progressive ratio responding for sucrose thereby increasing the motivation to obtain food (Davis et al., 2011) and in wild-type mice, leptin administration can decrease striatal dopamine D2 receptor binding (Pfaffly et al., 2010). This suggests that leptin reduces DA function within the striatum to reduce the motivation to obtain food. Leptin administration may decrease the motivation to seek food by decreasing the intrinsic excitability of VTA DA neurons (Hommel et al., 2006) or by decreasing the probability of glutamate release onto VTA neurons leading to presynaptic inhibition ultimately inhibiting appetitive behavior for rewarding stimuli (Thompson and Borgland, 2013). With leptin receptors showing increased sensitivity during the SD winter months, activity in the DA mesolimbic system would be significantly reduced thereby reducing food seeking behaviors.

Alternatively, because leptin receptors are expressed on VTA neurons that show a dense set of projections to the extended central amygdala (Leshan et al., 2010), it might also be feasible that increased sensitivity to leptin during the SD winter months more strongly activates activity in the central nucleus of the amygdala thereby resulting in anxiety-like behaviors (Hunter et al., 2007). This enhanced activity in the amygdala may then contribute to an enhanced HPA response leading to elevated circulating levels of glucocorticoids and ultimately decrease the propensity of the organism to seek reward or food in the environment.

## Conclusion

We entertained the possibility that seasonal changes in stress-or feeding-related hormones might dampen increase in reward-seeking behaviors. While it seems plausible that increased levels of glucocorticoids, as found in the winter, could interfere with DA output to stimulate food-seeking behaviors, the mechanism is convoluted. Alternatively, decreased levels of ghrelin or increased levels of leptin, as found in the winter, seem to be likely candidates in interfering with food-seeking behaviors in a DA-dependent mechanism. On the other hand, it seems just as likely that increased ghrelin activity or decreased leptin activity, as found in the summer, would work to facilitate an increase in food-seeking behavior in a DA-dependent fashion. As well, a combination of increased glucocorticoid activity, decreased ghrelin activity and increased leptin activity in the winter may impede DA-mediated change in food-seeking behaviors. Finally, other seasonal fluctuations in physiological processes (e.g., melatonin/serotonin tone), not covered in this review, could also play important roles in modulating seasonal effects on feeding behavior. While not being able to make any decisive conclusions on underlying neural mechanisms that mediate seasonal variation in food-related behaviors, we hope that this review serves to generate hypotheses for future experiments.

### Conflict of interest statement

The authors declare that the research was conducted in the absence of any commercial or financial relationships that could be construed as a potential conflict of interest.
